# Assessing the Suicidal Ideation and Its Associated Factors Among Undergraduate Medical Students and Residents in Iran: A Multi‐Center Cross‐Sectional Research

**DOI:** 10.1002/hsr2.71489

**Published:** 2025-11-10

**Authors:** Ahmad Nemati, Amir Hossein Jalalpour, Soodeh Jahangiri, Fatemeh Shaygani, Hafez Shojaadini, Deniz Naghibi, Razieh Amiri, Vida KardanMoghaddam, Milad Ahmadi Marzaleh

**Affiliations:** ^1^ Mashhad University of Medical Sciences Mashhad Iran; ^2^ Student Research Committee Shiraz University of Medical Sciences Shiraz Iran; ^3^ Health Policy Research Center, Institute of Health Shiraz University of Medical Sciences Shiraz Iran; ^4^ Endocrine and metabolism research institute Iran University of medical sciences Tehran Iran; ^5^ Department of Public Health Sciences, School of Medicine and Dentistry University of Rochester Rochester NY USA; ^6^ School of Medicine and Dentistry Griffith University Queensland Australia; ^7^ Department of Health in Disasters and Emergencies, Health Human Resources Research Center, School of Health Management and Information Sciences Shiraz University of Medical Sciences Shiraz Iran

**Keywords:** internship and Residency, Iran, students, suicidal ideation, suicide

## Abstract

**Background and Aims:**

Suicidal ideation (SI) is a serious concern among undergraduate medical students (UMSs) and residents worldwide. This study aimed to provide up‐to‐date evidence on SI and its determinants among this risky population across Iranian medical universities, in light of increasing sanctions and the resulting economic, cultural, and social challenges faced by Iran.

**Methods:**

In this cross‐sectional study, which was conducted from September 2023 to July 2024, UMSs and residents studying at Iranian universities were selected via convenience sampling. Participants completed an online 36‐item questionnaire containing demographic information and the Beck Scale for Suicidal Ideation, assessing suicidal thoughts over the past 6 months. Data were analyzed using SPSS software (version 25), with descriptive statistics and inferential analyses (Mann–Whitney U and Kruskal‐Wallis H tests), considering *p*‐value < 0.05.

**Results:**

Overall, 354 UMSs and 56 residents with a mean age of 23.6, and 30.6 years participated, respectively. About 50% of participants had suicidal thought, and the mean scores of SI among UMSs and residents were 10.3 ± 9.1, and 10.1 ± 7.6, in turn. Also, 12% of participants reported that they are prepared to commit suicide. Univariate analysis showed that age and gender were not significantly associated with BSSI scores. In both UMS and residents, higher SI was associated with sleep deprivation, burnout, doubts about continuing education, and substance use, while lower SI was associated with being married. Having paid employment was associated with lower SI only in residents. Being local to the university, financial stability, and religiosity were associated with lower SI exclusively among UMS.

**Conclusion:**

This study highlights a surprisingly high percentage of suicidal ideation and underscores its’ multifaceted nature. These results call for urgent attention to mental health support systems within medical education, particularly in light of the poor conditions of UMSs and residents.

**Trial Registration:** Not applicable.

## Summary

- Suicidal ideation is prevalent among medical students and residents globally.
- We found that more than three out of five Undergraduate Medical Students and Residents in Iran reported suicidal ideation or preparing to commit suicide.
- The findings highlight the urgent need for targeted mental health support systems in medical education to address the unique challenges faced by UMSs and residents in Iran.

**Abbreviations**
SI: Suicidal Ideation
SUD: Substance Use Disorder
UMSs: Undergraduate Medical Students

## 1 Background

Suicide is a major public health issue, with the total suicide rate of 9.2 worldwide per 100,000 people or more than 720 thousand individuals [1, 2]. Suicide has affected not only ordinary people, but also special groups, even physicians and medical staff [3, 4]. According to the American Medical Association Council on Medical Education report, medical students are three times more likely to die by suicide compared to their peers in the general population [5]. Also, suicide was reported as the second leading cause of death among medical residents in the USA in a study published in 2017 [6]. This rate, however, varies by region and context; for instance, a systematic review found that Suicidal Ideation (SI) among medical students ranges from 3.0% to 53.9% [7]. SI is influenced by various factors among Undergraduate Medical Students (UMSs) and medical residents, including high levels of stress, depression, hopelessness, anxiety, escalating family problems, lack of social supports, limited social interactions, exposure to traumatic situations, financial difficulties, and burnout [8]. Moreover, low salaries, extended work shifts exceeding high standards, along with perfectionism, and challenging working conditions contribute to higher levels of depression and burnout among residents [9, 10, 11], which, in turn, can lead to suicidal thoughts and behaviors [12]. Given these wide‐ranging figures, suicide is a global concern that requires evaluation within each country, as societal characteristics, cultural backgrounds, economic conditions, and factors related to education and work all play a significant role.

SI is particularly significant among Iranian UMSs and residents, and it has worsened in recent years [13]. Previous studies have shown that almost 16% of UMSs at two Iranian universities reported experiencing SI [14]. This rate was 34% for medical residents in Iran, indicating a critical situation of concern [5]. Evidence suggests that the suicide rate among medical residents in Iran is 10‐13 times higher than that of the general population (100 vs. 6.5 individuals per 100,000) [5]. According to a 2023 report by the Iran Medical Council, 16 residents tragically took their own lives over the course of a few months [5]. This is not only a tragic issue for human resources in the Iranian healthcare system but also equates to losing financial resources since the cost of education is mainly subsidized (more than 140,000 dollars for each medical student to graduate) [15]. When we take a look at the prevalence of mental illness among UMSs and medical residents in Iran, a recent meta‐analysis study revealed that depression and anxiety were reported among 43% and 44% of them, respectively [16]. Therefore, there might be some organizational problems in medico education system and some individual characteristics behind this phenomenon that make Iranian residents and UMSs susceptible to mental health conditions and SI.

The framework for medical education in Iran includes both public and private medical institutions, which are vital in shaping the training of future doctors. Also, medical program is a 7‐year undergraduate program involving theoretical courses along with extensive clinical training and internship in affiliated public hospitals [17]. Upon graduation with a Medical Doctor degree (M.D.), graduates have two options. They can start their practice as a doctor or compete in a highly competitive national residency exam. Those who pass this exam enter specialized residency programs ranging from three to 5 years, depending on the chosen field, such as internal medicine, surgery, or other specialties [18].

While important research on SI has been carried out in several cities and medical universities across Iran, the present study seeks to contribute significant insights into SI and its associated factors at a wide range of medical universities throughout the country. This can assist health policymakers and decision‐makers in urgently reforming harm‐reduction strategies for UMSs and residents across medical universities in Iran. Moreover, by leveraging the insights gained from this study, other countries can enhance their understanding of SI within their own medical communities, leading to more effective preventive strategies and support mechanisms tailored to their unique cultural contexts.

## 2 Methods

### 2.1 Study Design

This study, which was conducted from September 2023 to July 2024 in most medical universities throughout Iran, employed a cross‐sectional design to assess SI and its associated factors among UMSs and residents studying in Iran [19].

### 2.2 Participants and Sampling

**Inclusion criteria:** 1‐Participants eligible for this study included UMSs and medical residents who were currently enrolled in accredited medical universities in Iran during the study period. Specifically, UMSs in Iran enroll in the 7‐year undergraduate medical education program, which encompasses a comprehensive curriculum and an internship period, culminating in qualification as medical doctors or general practitioners. Medical residents in Iran are defined as medical doctors undertaking postgraduate specialty training programs, which typically last three to 5 years following completion of the undergraduate medical program. 2‐To ensure adequate exposure to the educational program and relevant experiences, all participants had completed at least 6 months of their respective training programs at the time of data collection.

**Exclusion criterion:** The only exclusion criterion was unwillingness to participate.

**Sample size:** Based on a report in 2023, the number of UMS students and residents studying in medical sciences universities in Iran was 92,453 [20]. Using the Cochran formula (n= $\frac{Nz^{2}\text{pq}}{Nd^{2}+z^{2}\mathrm{pq}}$), the sample size was calculated as 383 with a confidence level of 95% and an error of 5%.

**Sampling:** Participants were recruited using a convenience sampling approach. Invitations to participate were distributed via electronic questionnaire links shared through WhatsApp and Telegram groups and channels affiliated with medical universities in Iran. The invitation messages, including the questionnaire link, were sent to these platforms twice a month. This approach was implemented to expand outreach and continuously inform potential participants that data collection remained active until the desired sample size was not only met but exceeded.

### 2.3 Data Collection

The data collection tool in this study was an online questionnaire designed using Porsline website (An Iranian website for designing e‐questionnaires for conducting research). This 36‐item questionnaire divided into two sections: the participant demographics and characteristics (17 questions) and a Beck Scale for Suicidal Ideation (BSSI) (19 items) [21]. In this study, the translated, valid, and reliable version of BSSI was used (the reliability of this questionnaire has been reported as 0.95 in a previous study) [22].

In the survey introduction, the research objectives and criteria were clearly outlined to inform the potential participants, and it was stated that participation was voluntary and that they could withdraw at any time without providing a reason. If they were willing to participate in this study, they could start answering the questionnaire, just after checking the voluntary participation and informed consent item.

Next, the questionnaire started with participant demographics and characteristics, including gender, age, and marital status. Additionally, it inquired whether they continued to live in the same residence as before starting this level of education as well as geographical direction of university city and their year of study. The questionnaire also included an item assessing the perceived level of difficulty of the residency program which was only applicable to residents. The questionnaire also assessed whether participants held any paid jobs outside their roles as students, and whether their income was sufficient to cover their expenses. Other questions focused on self‐reported factors such as religiosity, sleep deprivation, burnout, interest in their field of study, and doubts about continuing their education. Participants also provided self‐reported data having ever been exposed to a traumatic situation, alcohol consumption (at least once in the past 6 months) and illicit drug use (at least once in the past 6 months).

It was followed by the BSSI to evaluate the presence and severity of SI among participants, which were scored based on a three‐point Likert‐type scale (ranging from 0 to 2). The total score for each participant is calculated by summing the individual scores, with a possible range from 0 to 38. The interpretation of the overall score is as follows: a score of 0 to 5 indicates the absence of suicidal thoughts, a score of 6 to 19 suggests the presence of suicidal thoughts, and a score of 20 to 38 indicates readiness to commit suicide [21]. Participants were asked to reflect on these questions over the past 6 months.

It is important to note that any questionnaires that were not fully completed or submitted were automatically excluded, leading to no missing data in the final set of submitted questionnaires.

### 2.4 Data Analysis

In this study, the data were analyzed using IBM SPSS Statistics software (version 25). Quality assurance was ensured through supervision of the process of data collection, data entry into the software, and data analysis. The mean score and standard deviation of SI were calculated. Descriptive statistics (frequency and percentage) for SI are presented in Table 1. The standard deviation is denoted as SD in the results. The Mann–Whitney U test (to compare two independent groups) and Kruskal‐Wallis H test (when comparing three or more independent groups) were used for univariate analysis due to the non‐normal distribution of the SI scores [23]. All tests were two‐sided and a *p*‐value < 0.05 was considered statistically significant, following conventional statistical practice [24].

**Table 1:** Participant demographics and key characteristics.

Variables	Undergraduate Medical Students	Residents
	Frequency (Percentage)	Frequency (Percentage)
**Age (*n*)**		
n < 20	28 (7.9)	0 (0)
20 ≤ n < 23	87 (24.6)	0 (0)
23 ≤ n < 26	163 (46)	2 (3.6)
26 ≤ n < 29	63 (17.8)	12 (21.4)
29 ≤ n < 32	11 (3.1)	23 (41.1)
32 ≤ n < 35	0 (0)	11 (19.6)
35 ≤ n < 38	2 (0.6)	7 (12.5)
38 ≤ n < 41	0 (0)	0 (0)
41 ≤ n	0 (0)	1 (1.8)
**Gender**		
Female	194 (54.8)	29 (51.8)
Male	160 (45.2)	27 (48.2)
**Marital status**		
Single	292 (82.5)	30 (53.6)
Married	62 (17.5)	26 (46.4)
**Local to the university location**		
Yes	151 (42.7)	19 (33.9)
No	203 (57.3)	37 (66.1)
**University location**		
Central	98 (27.7)	35 (62.5)
East	52 (14.7)	6 (10.7)
West	88 (24.9)	7 (12.5)
North	80 (22.6)	6 (10.7)
South	36 (10.2)	2 (3.6)
**Year of study**		
First	0 (0)	15 (26.8)
Second	15 (4.2)	22 (39.3)
Third	70 (19.8)	9 (16.1)
Forth	78 (22)	10 (17.9)
Fifth	103 (29.1)	0 (0)
Sixth	70 (19.8)	0 (0)
Seventh	18 (5.1)	0 (0)
**Level of difficulty of residency field**		
Less difficult	—	9 (16.1)
Moderately difficult	—	17 (30.4)
Very difficult	—	30 (53.6)
**Having other paid jobs**		
Yes	114 (32.2)	19 (33.9)
No	240 (67.8)	37 (66.1)
**Making ends meets (from those who have other jobs)**		
Yes	66 (57.9)	11 (57.9)
No	48 (42.1)	8 (42.1)
**Religiosity**		
Strong believer	87 (24.6)	9 (16.1)
Religious to some extent	155 (43.8)	24 (42.9)
Non‐religious	112 (31.6)	23 (41.1)
**Sleep deprivation**		
Yes	228 (64.4)	43 (76.8)
No	126 (35.6)	13 (23.2)
**Burnout**		
Yes	212 (59.9)	34 (60.7)
No	142 (40.1)	22 (39.3)
**Interest in the field of study**		
Yes	250 (70.6)	33 (58.9)
No	104 (29.4)	23 (41.1)
**Exposure to traumatic clinical situation**		
Yes	157 (44.4)	34 (60.7)
No	197 (55.6)	22 (39.3)
**Having doubts about continuing education**		
Yes	111 (31.4)	30 (53.6)
No	243 (68.6)	26 (46.4)
**Illicit drug use**		
Yes	50 (14.1)	15 (26.8)
No	304 (85.9)	41 (73.2)
**Alcohol use**		
Yes	113 (31.9)	29 (51.8)
No	241 (68.1)	27 (48.2)

### 2.5 Ethical Considerations

The proposal of this study was approved by the Ethics Committee affiliated with Shiraz University of Medical Sciences (SUMS), Shiraz, Iran, under the Ethical Approval (EA) code IR. SUMS. NUMIMG. REC.1402.151. Additionally, the study adhered to the ethical standards for medical research involving human subjects as outlined in the Declaration of Helsinki [25]. This study prioritized ensuring voluntary participation. Electronic Informed Consent (EIC) was obtained from each participant before they began to fill out the anonymous e‐questionnaire and they were informed that they could withdraw from the study without any justification.

## 3 Results

A total number of 354 UMSs and 56 residents from almost all medical schools in Iran (53 out of 58) participated in this study. Their mean ages were 23.6 (SD = 2.7) and 30.6 (SD = 3.3), and their female‐to‐male ratios were 1.2 and 1.07, respectively (Table 1). Moreover, a majority of UMS were in the 23‐26 age range (46%), while residents were primarily in the 29‐32 age range (41.1%). A significantly larger proportion of UMS were single (82.5%) compared to residents (53.6%).

As it can be seen from Table 2, the BSSI scores were similar between UMS (10.3 ± 9.1) and residents (10.1 ± 7.6). Approximately 50% of both UMS and residents reported experiencing suicidal thoughts (scores 6‐19). Moreover, more than 12% of both groups reported that they are ready to commit suicide.

**Table 2:** Frequency of suicidal ideation categories among the participants and comparison of the average score of SI between UMSs and residents.

Suicidal ideation categories	Undergraduate medical students	Residents
	Frequency (Percentage)	Frequency (Percentage)
Absence of suicidal thoughts (Score: 0 to 5)	131 (37)	21 (37.5)
Having suicidal thoughts (Score: 6 to 19)	179 (50.6)	28 (50)
Readiness to commit suicide (Score: 20 to 38)	44 (12.4)	7 (12.5)
BSSI Mean total score ± SD	10.3 ± 9.1	10.1 ± 7.6

Analysis of UMS and residents revealed distinct associations with SI, as measured by the BSSI (Table 3). While age and gender showed no significant correlation with BSSI scores in either group, several factors were linked to higher SI (*p*‐value > 0.05). Specifically, experiencing sleep deprivation and burnout, having doubts about continuing education, and engaging in illicit drug or alcohol use were associated with elevated BSSI scores in both UMS and residents. Conversely, being married and expressing interest in the field of study were associated with lower BSSI scores in both groups.

**Table 3:** Univariate analysis of demographic determinants of suicidal ideation.

Variables	Undergraduate Medical Students		Residents	
	BSSI Mean ± SD score	*p*‐value	BSSI Mean ± SD score	*p*‐value
**Age (n)**		0.70^a^		0.69^a^
n < 20	12.2 ± 11.4		—	
20 ≤ n < 23	10.6 ± 8.3		—	
23 ≤ n < 26	9.7 ± 8.9		12 ± 12.7	
26 ≤ n < 29	10.6 ± 9.9		9.08 ± 7.05	
29 ≤ n < 32	10.09 ± 8.8		11.3 ± 7.9	
32 ≤ n < 35	—		11.8 ± 8.6	
35 ≤ n < 38	10.5 ± 3.5		5.8 ± 4.1	
38 ≤ n < 41	—		—	
41 ≤ n	—		5	
**Gender**		0.92^b^		0.19^b^
Female	10.5 ± 9.5		11 ± 7.17	
Male	10.1 ± 8.6		9.2 ± 8.1	
**Marital status**		**0.003** ^b^		**0.04** ^b^
Single	11.09 ± 9.6		12.2 ± 8.4	
Married	6.8 ± 5.07		7.8 ± 5.9	
**Being a local of university location**		**0.001** ^b^		0.80^b^
Yes	8.1 ± 6.5		10.2 ± 7.8	
No	12 ± 10.4		10.1 ± 7.6	
**University location**		0.34^a^		0.35^a^
Central	9.4 ± 8.01		11.02 ± 7.6	
East	9.7 ± 8.9		5.6 ± 3.4	
West	9.8 ± 8.7		11.7 ± 9.2	
North	10.8 ± 9.6		6.3 ± 5.2	
South	13.9 ± 11.5		15 ± 14.1	
**Year of study**		0.55^a^		0.11
First	—		13.2 ± 7.1	
Second	11.3 ± 10.7		8.5 ± 6.1	
Third	10.9 ± 10.2		13 ± 10.2	
Forth	9.2 ± 8.4		6.8 ± 7.1	
Fifth	9.8 ± 8.6		—	
Sixth	11.9 ± 9.9		—	
Seventh	8.4 ± 5.2		—	
**Level of difficulty of residency field**		—		**0.006** ^a^
Less difficult	—		4.1 ± 3.5	
Moderately difficult	—		8.8 ± 6.3	
Very difficult	—		12.7 ± 8.08	
**Exposure to traumatic clinical situations**		0.12^b^		0.06^b^
Yes	11.4 ± 10.08		12.02 ± 8.3	
No	9.5 ± 8.2		7.3 ± 5.4	
**Having other paid jobs**		0.93^b^		**0.004** ^b^
Yes	10.3 ± 9.2		6.1 ± 5.7	
No	10.3 ± 9.1		12.2 ± 7.6	
**Making ends meets (from those who have other jobs)**		**0.005** ^b^		0.07^b^
Yes	8.04 ± 7.2		3.6 ± 2.1	
No	13.4 ± 10.8		7.9 ± 6.8	
**Religiosity**		**0.03** ^a^		0.40^a^
Strong believer	5.9 ± 5.4		8.2 ± 8.5	
Religious to some extent	9.8 ± 8.1		9.9 ± 8.1	
Nonreligious	14.5 ± 10.9		11.2 ± 6.8	
**Sleep deprivation**		**< 0.001** ^b^		**0.02** ^b^
Yes	11.4 ± 9.4		11.3 ± 7.5	
No	8.3 ± 8.2		6.3 ± 6.8	
**Burnout**		**< 0.001** ^b^		**0.001** ^b^
Yes	12.2 ± 9.6		12.8 ± 7.7	
No	7.5 ± 7.6		6.09 ± 5.3	
**Interest in the field of study**		**0.005** ^b^		**< 0.001** ^b^
Yes	9.6 ± 8.7		7.2 ± 6.6	
No	12.1 ± 9.8		14.4 ± 6.9	
**Having doubts about continuing education**		**< 0.001** ^b^		**0.001** ^b^
Yes	14.5 ± 10.9		13.1 ± 7.3	
No	8.4 ± 7.4		6.7 ± 6.5	
**Illicit drug use**		**< 0.001** ^b^		**0.003** ^b^
Yes	21.7 ± 12.1		15.06 ± 6.9	
No	8.4 ± 7.02		8.3 ± 7.1	
**Alcohol use**		**< 0.001** ^b^		**0.03** ^b^
Yes	16.3 ± 11.5		12.2 ± 7.7	
No	7.5 ± 6.01		8 ± 7	

Significant values (*p*‐value < 0.05) are in **bold**.

a Kruskal‐Wallis test

b Mann‐Whitney test

Furthermore, having other paid jobs was associated with lower BSSI scores only in residents, not in UMS (UMS: 10.3 ± 9.2 vs. 10.3 ± 9.1; Residents: 6.1 ± 5.7 vs 12.2 ± 7.6). On the other hand, being local to the university location, managing to make ends meet, and practicing religiosity were only associated with lower BSSI scores exclusively among UMS.

## 4 Discussion

Our study revealed that only about 37% of both medical residents and UMSs reported no suicidal thoughts. The remaining participants had either experienced suicidal ideation or had considered acting on those thoughts (Table 2). The comparable mean scores—10.3 for UMSs and 10.1 for residents, indicates that both groups face comparable pressures and challenges, despite being at different stages in their academic and professional journeys. Previous systematic reviews have reported global SI rates among UMSs ranging from 1.8% to 53.6% [26], with another review estimating rates between 3% and 53.9% [7]. In Pakistan [27] and Saudia Arabia [28] (two neighboring countries of Iran) approximately one out of three UMSs had SI. While among Dutch medical residents, the prevalence of SI was noted to be 12% [29]. Prevalence of SI across Iranian general population was 13% [30, 31], while this rate was higher in Iranian UMSs and residents as the prevalence of SI among UMSs in southern Iran was reported at 24.7% in 2022 [32]. Another evaluation in Yazd, in central Iran, indicated a rate of 16% for SI among UMSs [33]. In Tehran, the most populated city of Iran, a study found 34.3% of medical residents experience suicidal thoughts, based on the BSSI [34]. Our findings reveal an even higher prevalence than previous studies in Iran and surrounding countries, raising alarms about this significant issue.

Our study found no significant differences in age or year of study between participants with and without SI, consistent with findings from similar studies on medical residents in Iran [34]. Also, a worldwide review study highlighted that while depressive symptoms tend to increase over the course of undergraduate medical training, the prevalence of SI among UMSs did not vary significantly by the year of study [35]. Furthermore, gender was not associated with SI in our study and this aligns with a review of 17 global studies, where most showed no significant gender differences (12 reported no significance and four found higher rates in females) [26]. Similarly, in Northwest Ethiopia and Pakistan, female UMSs were found to have higher odds of SI and suicide attempts [27, 36, 37]. In contrast, a study of Japanese residents found that high‐risk male trainees and physicians were more likely to experience suicidal thoughts (31.2% vs. 22.1%) [38]. Similarly, across Iranian general population there was not differences of SI rate in males and females [31]. Among Iranian medical residents, gender was not a significant factor [34]; however, female UMSs exhibited higher rates of SI [33]. These varied findings in different countries suggest that societal factors and cultural context significantly shape these outcomes, warranting further investigation.

In our research, being married appeared to be associated with lower mean BSSI scores across medical residents and UMSs. This trend is consistent with the findings of several studies on UMSs and residents in other countries [39]. This finding in line with recent research in Iran and neighboring countries (e.g. in Turkey [40]), revealing that UMSs with single marital status face higher risks of SI [32, 33, 34]. This suggests that being married might be a protective factor against SI in residents and UMS, since it can play a crucial role in supporting mental health. Unfortunately, over the past few decades, economic conditions in Iran have contributed to an increase in the average age of marriage and a decline in marriage rates [41]. This may be another factor that may have an impact on the incidence of SI in recent years in Iran. The clinical implication of this finding underscores the importance of social support for medical students and residents.

A surprising finding in our study was that UMSs who were studying in their hometowns reported significantly lower SI scores (8.1 vs 12, *p*‐value = 0.003). The reason might be the emotional and financial support that UMSs receive from family in comparison to the UMSs who live away from home [42], and this might play as a protective factor against SI. In contrast, other studies in Iran did not find an association [33, 43]; however, it is important to note that our study had a larger sample size compared to previous studies (208 and 194 in comparison with 354 UMSs in our study). In terms of experiencing difficulty, residents who rated their residency field as “Very difficult” had significantly higher SI scores compared to those who described it as “Less difficult” (12.7 vs. 4.1). This aligns with studies conducted on Egyptian and Japanese medical residents [37, 38]. Moreover, the prevalence of SI and pressures associated with residency programs can vary in different specialties [37], but our study did not assess the specific specialties of the residents.

Interestingly, our study indicated that residents with part‐time jobs had lower SI scores compared to those without any additional employment (12.2 vs. 6.1; *p*‐value = 0.004). These findings are consistent with those reported in a study from Japan [38]. However, it is worth noting that Iranian residents are prohibited from working in the medical fields during their residency, which may add to their financial stress [5]. Besides, due to working long hours, most residents do not have sufficient time to seek additional sources of income in Iran [5, 9]. Additionally, the current economic condition in Iran, characterized by sanctions, currency devaluation, and financial hardship [44], while having a part‐time job may provide some financial relief.

Those UMSs and residents who experienced sleep deprivation had high BSSI scores, as Fan et al. reported that sleep deprivation significantly impacts the mental health of UMSs and can make them susceptible to SI [45]. It is crucial to implement strategies that optimize sleep patterns and address the rigorous schedules associated with residency training. Our study also showed that UMSs experiencing burnout had significantly higher BSSI scores, and this aligns with our surrounding countries as Iraq [46] and India [47]. Regarding the high prevalence of burnout in Iranian UMSs and residents (around one out of four individuals) [48], it is recommended to inform educational institutions and healthcare organizations about the critical need for supportive measures aimed at reducing burnout among medical students and residents.

Furthermore, lack of interest in their chosen field and had doubts about continuing their medical education also showed significantly higher SI scores. It is of concern that only half of Iranian UMSs pursued their field of study based on personal interest, while the other half were motivated by familial pressure or societal expectations [49]. When these students face difficulties, such as low salaries or challenging academic demands, they are at an increased risk for SI and potentially dropping out of their programs. Similarly, Torres et al. have reported that a higher mean score of SI is associated with thoughts of abandoning the course [50]. This finding emphasizes the importance of career guidance and support for students who are experiencing uncertainty about their future in medicine.

Moreover, our findings indicated that substance use, including alcohol and illicit drugs, was associated with higher BSSI scores. Substance use disorder (SUD) can exacerbate mental health issues, creating a vicious cycle where individuals resort to substances as coping mechanisms [51]. Surprisingly in Pakistan, SUD was found to be the greatest risk factor for SI in UMSs [27], also the association of SUD and SI been observed in UMSs and residents globally [7]. Regarding Iranian general population, those who reported SUD were more susceptible to SI [52] and this was found in Iranian residents who reported higher rates of SI linked to substance use [34]. Unfortunately, it is estimated that prevalence lifetime of SUD is significantly high in Iranian UMSs (between 30% to 50%) [53] and this highlights the urgent need for comprehensive substance use prevention and intervention programs within medical education and training to address both mental health and substance use disorders effectively.

### 4.1 Limitations

A key limitation of this study is the potential for selection and response bias inherent in web‐based data collection, which may affect the representativeness and generalizability of the findings. Additionally, the cross‐sectional design restricts our ability to draw causal inferences between the identified factors and SI, as it remains unclear whether these factors contribute to SI or if SI influences these factors. While we utilized non‐parametric tests (Mann–Whitney U and Kruskal‐Wallis H) to identify significant associations, we chose not to employ logistic regression analysis due to concerns about meeting its assumptions, the exploratory nature of our study, and the relatively small sample size for residents, which could limit the reliability of such analyses. These limitations should be considered when interpreting our results, and future research could benefit from longitudinal designs and larger samples to further explore these relationships.

## 5 Conclusion

This study confirms a significant prevalence of suicidal ideation among Iranian undergraduate medical students and residents, underscoring the considerable mental health challenges faced by this population. This study highlights several critical factors associated with SI, like sleep deprivation, burnout, and substance use should. The unique stressors faced by UMSs, such as financial instability and academic doubts, should be mitigated through scholarship programs, mentorship initiatives, and career counseling services. For residents, the burden of demanding workloads, challenging fields, and financial strain from additional employment necessitates improved working conditions, fair compensation, and readily available mental health support tailored to their specific needs. This study emphasizes the need for proactive measures to support the mental well‐being of UMSs and residents. By fostering an environment that prioritizes mental health, educational institutions can help reduce the incidence of suicidal ideation and improve overall student well‐being.

## Human Ethics and Consent to Participate Declarations

The study proposal was approved by ethics committee of Shiraz University of Medical Sciences encoded IR. SUMS. NUMIMG. REC.1402.151. Informed consent was obtained from all subjects. Before conducting each interview, all participants were given sufficient information regarding the study objectives, and written consent was obtained from them. The interviewees were assured that the interviews would remain confidential, and audio files and notes would be saved anonymously. They were also informed that they could withdraw from the study at any stage they wish.

## Author Contributions

**Ahmad Nemati:** conceptualization, methodology, data curation, writing – original draft, writing – review and editing. **Amir Hossein Jalalpour:** writing – original draft, validation, formal analysis, supervision, conceptualization. **Soodeh Jahangiri:** conceptualization, writing – original draft, validation, formal analysis, supervision. **Fatemeh Shaygani:** conceptualization, writing – original draft, visualization, writing – review and editing, formal analysis, data curation, supervision. **Hafez Shojaadini:** supervision, data curation, validation, visualization, writing – review and editing, writing – original draft, conceptualization. **Deniz Naghibi:** conceptualization, writing – original draft, investigation, methodology, writing – review and editing, supervision, data curation. **Razieh Amiri:** data curation, supervision, writing – original draft, conceptualization, investigation, methodology, writing – review and editing. **Vida Kardan Moghaddam:** conceptualization, investigation, writing – original draft, writing – review and editing, methodology, data curation. **Milad Ahmadi Marzaleh:** conceptualization, writing – original draft, writing – review and editing, methodology, data curation, supervision, visualization.

## Conflicts of Interest

The authors have no conflict of interests to declare. The authors declare that Shiraz University of Medical Sciences provided financial support for this study. However, they had no role in the study design, data collection, analysis, interpretation of data, writing of the report, or decision to submit the report for publication.

## Consent to Participate

Informed consent was obtained from all subjects. Before conducting each interview, all participants were given sufficient information regarding the study objectives, and written consent was obtained from them. The interviewees were assured that the interviews would remain confidential, and audio files and notes would be saved anonymously. They were also informed that they could withdraw from the study at any stage they wish.

## Consent for Publication

The authors have nothing to report.

## Transparency Statement

The lead author Milad Ahmadi Marzaleh affirms that this manuscript is an honest, accurate, and transparent account of the study being reported; that no important aspects of the study have been omitted; and that any discrepancies from the study as planned (and, if relevant, registered) have been explained.

## Data Availability

The data that support the findings of this study are available from the corresponding author upon reasonable request. The datasets used and analyzed during the current study available from the corresponding author on reasonable request.
